# Current and future perspectives on functional molecular imaging in nephro-urology: theranostics on the horizon

**DOI:** 10.7150/thno.58682

**Published:** 2021-04-07

**Authors:** Yoshitaka Toyama, Rudolf A. Werner, Camilo A. Ruiz-Bedoya, Alvaro A. Ordonez, Kei Takase, Constantin Lapa, Sanjay K. Jain, Martin G. Pomper, Steven P. Rowe, Takahiro Higuchi

**Affiliations:** 1 Department of Nuclear Medicine, University Hospital Wuerzburg, Wuerzburg, Germany.; 2 Department of Diagnostic Radiology, Tohoku University, Sendai, Japan.; 3 Comprehensive Heart Failure Center, University Hospital Wuerzburg, Wuerzburg Germany.; 4 Division of Nuclear Medicine and Molecular Imaging, The Russell H. Morgan Department of Radiology and Radiological Science, Johns Hopkins University School of Medicine, Baltimore, MD, USA.; 5 Center for Infection and Inflammation Imaging Research, Johns Hopkins University School of Medicine, Baltimore, Maryland.; 6 Department of Pediatrics, Johns Hopkins University School of Medicine, Baltimore, Maryland.; 7 Nuclear Medicine, Medical Faculty, University of Augsburg, Augsburg, Germany.; 8 The James Buchanan Brady Urological Institute and Department of Urology, Johns Hopkins University School of Medicine, Baltimore, MD, USA.; 9 Sidney Kimmel Comprehensive Cancer Center, Johns Hopkins University School of Medicine, Baltimore, MD, USA.; 10 Graduate School of Medicine, Dentistry and Pharmaceutical Sciences, Okayama University, Okayama, Japan.

**Keywords:** Glomerular filtration rate, renal, kidney, renal function, positron emission tomography, nephrology, urology, molecular imaging, theranostics

## Abstract

In recent years, a paradigm shift from single-photon-emitting radionuclide radiotracers toward positron-emission tomography (PET) radiotracers has occurred in nuclear oncology. Although PET-based molecular imaging of the kidneys is still in its infancy, such a trend has emerged in the field of functional renal radionuclide imaging. Potentially allowing for precise and thorough evaluation of renal radiotracer urodynamics, PET radionuclide imaging has numerous advantages including precise anatomical co-registration with CT images and dynamic three-dimensional imaging capability. In addition, relative to scintigraphic approaches, PET can allow for significantly reduced scan time enabling high-throughput in a busy PET practice and further reduces radiation exposure, which may have a clinical impact in pediatric populations. In recent years, multiple renal PET radiotracers labeled with ^11^C, ^68^Ga, and ^18^F have been utilized in clinical studies. Beyond providing a precise non-invasive read-out of renal function, such radiotracers may also be used to assess renal inflammation. This manuscript will provide an overview of renal molecular PET imaging and will highlight the transformation of conventional scintigraphy of the kidneys toward novel, high-resolution PET imaging for assessing renal function. In addition, future applications will be introduced, e.g. by transferring the concept of molecular image-guided diagnostics and therapy (theranostics) to the field of nephrology.

## Introduction

**Figure 1:**
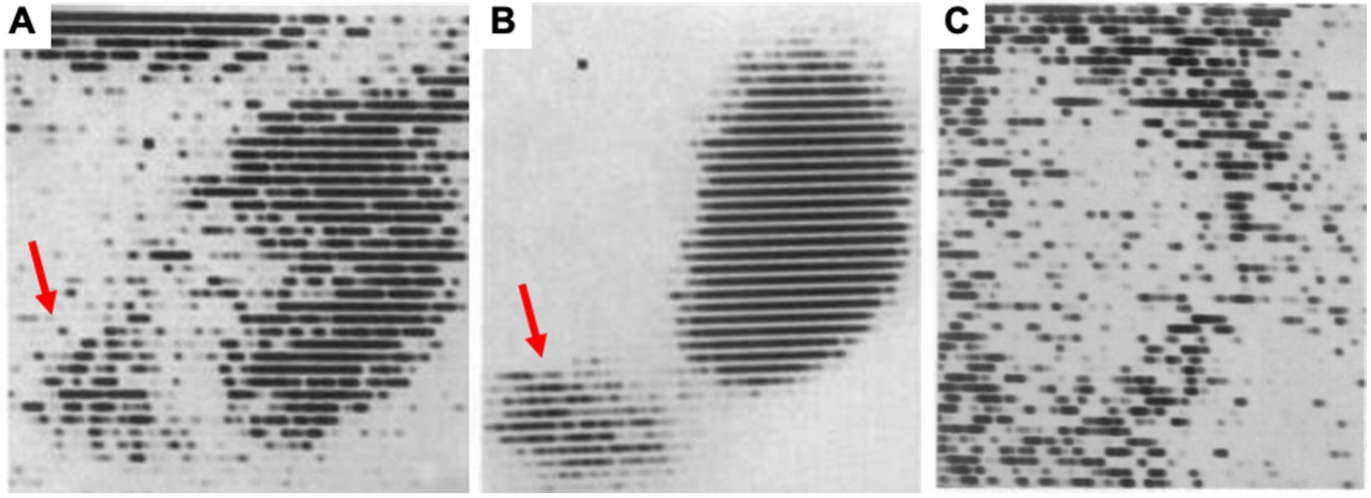
** First renal scintigraphy with Chlormerodrin (^203^Hg),** showing **(A)** rather less clear images shortly after transplantation, while in a long-term follow-up after 63 days **(B)**, a “great improvement in the renal contours“ was noted by Figueroa et al 1. Red arrows indicate uptake in the bladder. In **(C)**, a renal scan of patient 5 days after transplantation is displayed and the scan showed “negative contrast” with almost no uptake in the kidney. The patient died 8 days later. Adapted with permission from 1, copyright 1965 NEJM Massachusetts Medical Society.

**Figure 2:**
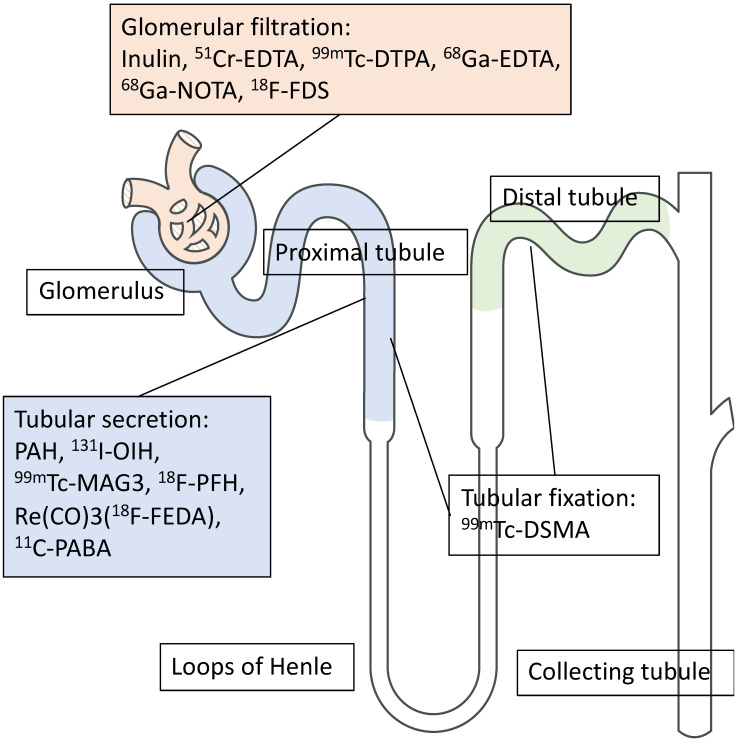
** Different mechanisms of renal radiotracer uptake and excretion.** EDTA: ethylenediaminetetraacetic acid, DTPA: diethylenetriaminepentaacetic acid, NOTA: 1,4,7-Triazacyclononane-1,4,7-triacetic acid, FDS: 2-deoxy-2-fluoro-D-glucose, PAH: p-aminohippurate, OIH: orthoidodohippurate, MAG3: mercaptoacetyltriglycine, Re(CO)3FEDA: Re(CO)3-N-(fluoroethyl)iminodiacetic acid, PABA: para-aminobenzoic acid, DMSA: dimercaptosuccinic acid.

Renal functional imaging with single-photon-emitting radiotracers has been used as a noninvasive diagnostic technique since the mid-1960s 1, and, during that time, three categories for renal radionuclide scintigraphy have been established (**Figure 1**). These are (1) radiotracers that are mainly filtered by the glomerulus to monitor glomerular filtration rate (GFR); (2) radiotracers that undergo tubular secretion for the measurement of effective renal plasma flow (ERPF); and (3), radiotracers that are taken up into the tubules so that morphological and/or tubular uptake function can be assessed (**Figure 2**, **Table 1**) 2.

Over the last decades, other imaging modalities for assessing renal function beyond renal radionuclide imaging have emerged and include, but are not limited to, ultrasound, computed tomography (CT), and magnetic resonance imaging (MRI). Ultrasound provides a fast and easily obtainable read-out of kidney morphology, while computed tomography (CT)-angiography provides in-depth renal vascular information. Renal MRI (rMRI), however, provides information with superior soft-tissue contrast to characterize renal masses or parameters on renal flow 3, 4. Due to the increased use of those imaging modalities, the use of renal scintigraphy has significantly decreased (approximately 25%) in the last decade 5, 6. Given its unique feature to reliably assess spilt renal function, renal scintigraphy is still frequently requested by referring physicians, especially for pediatric populations or for evaluating living donors for kidney transplantation 7. **Table 2** provides an overview of advantages and disadvantages for different imaging modalities used for renal functional imaging.

Recent years have also witnessed a shift from single-photon emission computed tomography (SPECT) to higher-resolution positron emission tomography (PET), which is supported by the overwhelming success and increased availability of PET imaging for oncological applications 8. Of note, various PET radiotracers have been evaluated to assess renal function, such as ^68^Ga-ethylenediaminetetraacetic acid (^68^Ga-EDTA), ^11^C-*para*-aminobenzoic acid (^11^C-PABA) and 2-deoxy-2-[^18^F]fluoro-D-sorbitol (^18^F-FDS) 9-15. In addition, the field of renal nuclear imaging has been further expanded by assessing urinary tract infections 16. The resulting refinements relative to SPECT may find growing applications for such highly specific renal PET-based imaging approaches. This review article will provide an overview of renal molecular PET imaging and will highlight the transformation of conventional scintigraphy of the kidneys toward novel, higher-resolution PET imaging for assessing renal function. In addition, future applications in clinical practice will be introduced, e.g. by applying non-invasive systemic read-outs for assessing kidney-organ crosstalk or by transferring the concept of molecular image-guided diagnostics and therapy (theranostics) from oncology to the field of nephrology.

### Dynamic renal scintigraphy with single-photon-emitting radiotracers

The most important quantitative characteristics for renal function are GFR and ERPF, and the gold standard for measuring both biomarkers is the clearance of exogenous substances, such as inulin and *para*-aminohippuate. Given the expensive and time-consuming nature of these diagnostics tests, they are nowadays seldomly performed. Normally, the kidneys receive 20% of cardiac output, with renal plasma flow averaging 600mL/min. The kidneys clear waste products and other substances from the plasma, and their clearances are determined by both glomerular filtration and tubular secretion. GFR is defined as the volume of filtrate by both kidneys per minute and is normally approximately 125 mL/min, i.e. 20 % of renal plasma flow delivered to the kidneys. A decline in GFR to <60 mL/min/1.73 m2 for ≥3 months is a common criterion for defining chronic kidney disease and such a loss in GFR has been well associated with a higher risk of all-cause mortality 17. In current clinical practice, GFR is estimated using serum creatinine, but that approach lacks precision 18, 19. ^51^Cr-labeled ethylenediaminetetraacetic acid (EDTA) and^ 125^I-labeled iothalamate are radiolabeled exogenous substances that offer more precise measurements of GFR, but are rarely used in currently clinical practice because of high costs and inability to provide split renal function 9, 11, 20. On the other hand, the true renal plasma flow cannot be calculated because no substance undergoes 100% first-pass extraction. The clearance of substances that are almost completely extracted include *para*-aminohippuate (PAH) or *ortho*-iodohippurate (OIH), which are 20% filtered by the glomerulus and 80% secreted in the tubules. As a result, effective renal plasma flow (ERPF) is used as a biomarker in clinical practice 21, 22. There is no blood marker to estimate ERPF, and clearance of PAH or radiolabeled OIH was used to measure ERPF, but similar to inulin clearance in GFR, this approach is not well established in clinical practice 9.

Instead of blood clearance of exogenous substances, dynamic renal scintigraphy with ^99m^Tc-diethylenetriaminepentaacetic acid (^99m^Tc-DTPA; GFR radiotracer) and ^99m^Tc-mercaptoacetyltriglycine (^99m^Tc-MAG3; ERPF radiotracer) is routinely performed to provide comprehensive information about the kidney. Due to its high accumulation and low background accumulation, ^99m^Tc-MAG3 allows for high contrast renal images 2, 18. ^99m^Tc-DTPA can provide an assessment of GFR because of its nature of radiotracer filtration at the glomerulus and minimal tubular uptake 2. The advantages of such renal scintigraphy include measurement of renal function along with comprehensive assessment of the entire urinary excretion system, including both kidneys and ureters. Furthermore, these techniques can be used in patients with poor renal function who are not eligible for CT-based contrast enhanced imaging. As a major advantage, radiation exposure is lower relative to CT, rendering these techniques as attractive kidney functional read-outs in pediatric populations, in particular for repeated renal studies 23.

As a major drawback, renal scintigraphy has only limited spatial resolution of > 1cm when compared with other imaging modalities. Assessment is mainly based on planar dynamic images. Compton scatter and soft-tissue attenuation can further reduce the image resolution as well as quantification reliability 9, 11. The combination of SPECT and CT (SPECT/CT) can be used for soft tissue attenuation correction using CT attenuation map 9, 11, although this adds significantly to the radiation exposure and lacks the temporal resolution of planar imaging. The recently introduced high-end cadmium-zinc-telluride (CZT) cameras have even better energy resolution which may further reduce scatter artifacts 24.

### Comparison of PET and conventional renal nuclear imaging

In contrast to scintigraphy with single-photon-emitting radiotracers, PET provides multiple advantages including high temporal resolution and sensitivity allowing dynamic three-dimensional acquisition, superior spatiotemporal resolution, absolute quantification with radiotracer kinetics modeling and three-dimensional co-registration with CT images. PET imaging for assessment of the urinary tract using standard scanners with an axial field of view of 20 cm cannot cover the entire urinary system in a single field of view for a continuous three-dimensional dynamic san 9, 25. Recently introduced large axial view PET scanners such as the total-body PET Explorer, however, can image the entire body with one bed position (axial field of view 194 cm) and thus, such novel scanners may allow for imaging the entire urinary tract using dynamic acquisition 26.

### Positron emitting radionuclides

Various positron-emitting radionuclides have been used clinically and their characteristics are defined by different half-lives of the isotope, positron range, and production source (**Table 3**). ^18^F and ^11^C, recognized as common standard PET isotopes, are synthesized by a cyclotron, whereas ^68^Ga is typically produced by a generator. Longer half-life of ^18^F (110 min) is a major advantage as it allows radiotracer delivery from central cyclotron facilities and flexibility in study design in clinical practice (e.g. delayed imaging protocols). Because of its shorter half-life (20min), ^11^C-labeled radiotracers are difficult to use in clinical routine 27. On the other hand, ^68^Ga has a relatively longer half-life (68 min) but it requires an in-house ^68^Ge/^68^Ga generator 28. Since ^68^Ga-labeled radiotracers for somatostatin-receptor (SSTR) and prostate-specific membrane antigen (PSMA) imaging for neuroendocrine tumors and prostate cancer are frequently used in the clinic, the availability of such generators has improved 29. ^68^Ga, however, has a relatively high positron energy with long positron range when compared to ^18^F which results in reduced spatial resolution 28, 29. Moreover, in nuclear oncology, there is a shift from ^68^Ga to ^18^F-labeled radiotracers and one may speculate that this trend will also further fuel the development and increased clinical use of ^18^F radiotracers for renal radionuclide imaging 8.

### GFR agents

#### 68Ga-ethylenediaminetetraacetic acid (EDTA)

^68^Ga-EDTA reflects GFR, and is the only renal imaging agent to date that has been evaluated in a large clinical trial. Of note, ^68^Ga-EDTA has been validated since the 1960s, before the widespread use of PET 30, 31. Physiologically stable metal chelates like EDTA are excreted by glomerular filtration and, until recently, were widely used for the quantitative assessment of GFR by using ^51^Cr-EDTA. Later, with the development of gamma cameras, ^99m^Tc-labeled renal radiotracers became the main focus of research. With the increased availability of PET imaging in the past two decades, ^68^Ga-based PET imaging of the kidneys has reemerged 11.

Hicks et al. first revealed the excellent kinetics of ^68^Ga-EDTA as a renal radiotracer in small animal studies 32, and then investigated the agent in humans 10. ^68^Ga-EDTA initially concentrates in the blood pool, promptly begins to accumulate in the renal cortex, transfers to the renal parenchyma and further into the collecting system 3-4 minutes after administration. In a study of 31 patients with different degrees of kidney function, ranging from severely impaired to normal, Hofman et al. calculated GFR from PET images of ^68^Ga-EDTA as well as compared the results with GFR from ^68^Ga-EDTA and ^51^Cr-EDTA plasma sampling. The GFR measured by ^68^Ga-EDTA and ^51^Cr-EDTA plasma sampling showed an excellent correlation (Pearson correlation coefficient of 0.94). GFR quantified by ^68^Ga-EDTA clearance derived from three organ compartments including the kidneys, ureter and the bladder correlated well with GFR from plasma sampling (correlation co-efficient 0.9). In subjects with a ^51^Cr-EDTA GFR of more than 150 mL/min, ^68^Ga-GFR was significantly lower and the authors explained such findings by plasma binding, tubular secretion, or dissociation *in vivo*. Furthermore, in 11 patients, ^99m^Tc-dimercaptosuccinic acid (^99m^Tc-DMSA) was conducted and read-out included a renal static scintigraphy approach, planar imaging, and SPECT/CT. Good agreement between ^99m^Tc-DMSA SPECT/CT and excretory phase (2-10 minutes) from ^68^Ga-EDTA dynamic PET was noted. The latter imaging modality, however, allows more in-depth information on the entire urinary tract system in a 3-D capability relative to SPECT 10, 11.

#### ^68^Ga-1,4,7-Triazacyclononane-1,4,7-triacetic acid (^68^Ga-NOTA)

^68^Ga-NOTA is a ^68^Ga-labeled metal chelate whose clearance reflects GFR, similar to ^68^Ga-EDTA. Lee et al. measured serum protein binding of ^68^Ga-NOTA and performed small animal PET studies for comparison with ^51^Cr-EDTA. ^68^Ga-NOTA showed the lowest serum protein binding compared to the other ^68^Ga-labeled chelating agents (DOTA, DTPA and EDTA). Furthermore, both ^68^Ga-NOTA and ^51^Cr- EDTA showed very similar biodistribution patterns and almost the same GFR 33. Although it is likely that their results can be replicated in humans, further studies in patients are needed.

#### ^68^Ga-IRDye800-tilmanocept

Another GFR-reflecting agent, radiolabeled tilmanocept has been reported as a unique radiotracer for evaluating diabetic nephropathy. The number of diabetic patients continues to increase, with diabetic nephropathy being the leading cause of chronic renal failure and occurring in 40% 34. In such patients, nephropathy progresses slowly over 20 to 30 years, with no symptoms or decline in kidney function in early stages. GFR decline occurs predominantly in advanced nephropathy, which highlights the urgent need to develop imaging tests providing reliable information on the progression of the disease. Qin et al. investigated radiolabeled tilmanocept imaging in mice and its colocalization in the glomerulus with histologic examination. PET imaging of ^68^Ga-IRDye800-tilmanocept demonstrated receptor-mediated renal accumulation with evidence of glomerular uptake. Histologic examination demonstrated colocalization of CD206 which resides on the surface of mesangial cells, and IRDye800-tilmanocept within the glomerulus. Furthermore, molecular imaging of diabetic and nondiabetic mice was performed with fluorescence-labeled ^99m^Tc-tilmanocept, and hyperfiltration of tilmanocept was observed in diabetic mice. Given the important role of mesangial cells during the progression of diabetic nephropathy, PET or SPECT renal imaging with radiolabeled tilmanocept may provide noninvasive quantitative assessment of glomerular function even in early disease stages 35.

#### ^18^F-FDS

^18^F-FDS, which is a sorbitol derivative filtered through the glomerulus, has shown excellent pharmacokinetics and is readily available by a simple one-step synthesis from the most commonly used radiotracer ^18^F-FDG. This makes it available at virtually every PET practice with access to, or supply by, a cyclotron facility 36. In early human studies, urinary clearance of sorbitol was reported to be identical to that of inulin (sorbitol-to-inulin clearance ratio = 1.01) 37. Hence, ^18^F-FDS, an analogue of sorbitol, may have similar kinetics as inulin, with free filtration at the renal glomerulus and no tubular reabsorption.

To explore the potential of ^18^F-FDS as a renal PET radiotracer, basic biodistribution properties and clearance through the renal collecting system pathway were explored in healthy rats. Dynamic PET imaging showed rapid accumulation in the kidneys followed by a time-dependent transition to the collecting system. There was almost no accumulation of background, such as liver, intestines and spleen, suggesting low hepatobiliary clearance and exclusive renal filtration of ^18^F-FDS. PET renography showed cortical accumulation in the initial blood flow phase and subsequent rapid parenchyma and transfer of the radiotracer to the collecting system. Split renal function assessment demonstrated a normal renogram pattern. Accumulation in the cortex in the early blood flow phase was confirmed by *ex vivo* autoradiography. Comparable renal distribution of ^18^F-FDS and ^99m^Tc-DTPA was achieved in the identical animal after dual radiotracer administration. Postmortem tissue analysis showed that ^18^F-FDS had the highest accumulation in the kidneys at all imaging time points. Radio-thin-layer chromatography analysis also demonstrated that no radioactive metabolites were detected in blood and urine 35 minutes after radiotracer administration. Furthermore, *in vivo* serum protein binding of ^18^F-FDS was less than 0.1% at 35 minutes after radiotracer administration 13. Only the free fraction of the radiotracer is filtered at the glomerulus and protein binding in the blood leads to underestimation of the GFR. The protein binding of ^99m^Tc-DTPA has been reported to range from 2% to 10% 38, 39 and the 100-fold lower serum binding properties of ^18^F-FDS indicate that ^18^F-FDS is not only an excellent GFR substance with excellent pharmacokinetics similar to the gold standard inulin, but also has a substantially higher stability in the blood relative to its SPECT counterpart.

Based on these preclinical results, the potential of ^18^F-FDS as a functional renal imaging agent has been investigated using two different rat models of acute renal failure (ARF) and unilateral ureteral obstruction (UUO). On PET images, ARF rats, induced by intramuscular injection of glycerol, had significantly less uptake in the renal cortex and slower excretion into the bladder than controls. Furthermore, the renogram presented a non-functional pattern. In UUO rats, obtained by complete ligation of the left ureter near the renal pelvis, lower and delayed uptake was observed on the obstructed left side, with no transition into the collecting system, whereas the contralateral side showed results similar to those obtained in control rats. Renograms showed that the UUO collecting system presented a typical obstructed pattern 15. These results indicate the potential of ^18^F-FDS as a radiotracer providing a non-invasive read-out of the urinary tract that may also allow for precise GFR measurements.

**Figure 3:**
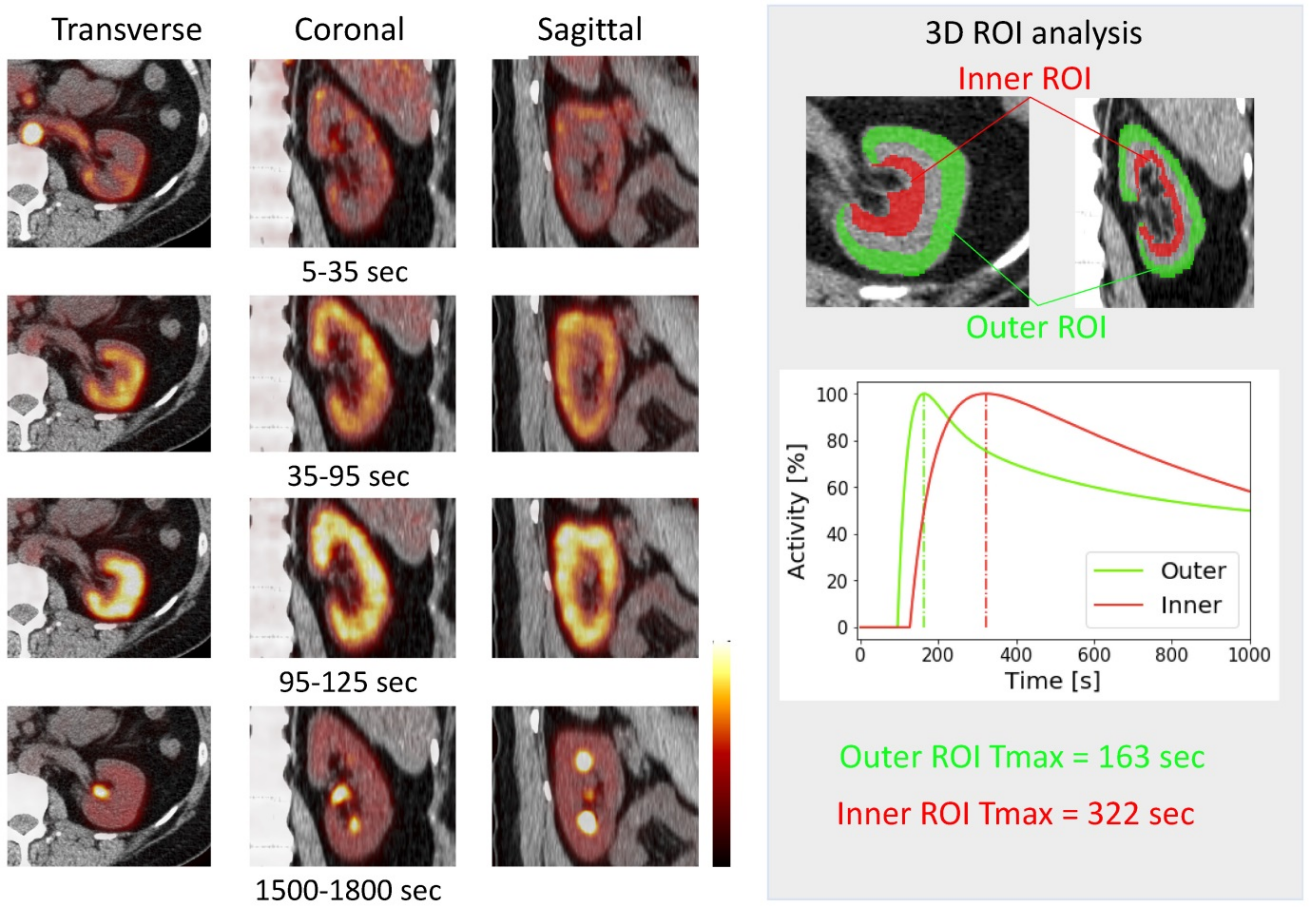
** First *in-vivo*^18^F-Fluorodeoxysorbitol (^18^F-FDS) images in a healthy volunteer.** Dynamic axial, coronal and sagittal images (left) showed rapid radiotracer accumulation in the renal cortex. A time-activity curve (right) obtained by dynamic PET imaging. Three-dimensional volumes of interest were placed on the outer layer (corresponding to the cortex, green) as well as inner layer (red) corresponding to the medulla 14.

Dynamic ^18^F-FDS PET was performed in two healthy human volunteers 14. The renal cortex was first delineated and activity gradually transited in the parenchyma, followed by radiotracer excretion (**Figure 3**). The kidneys had the highest radiotracer accumulation after the initial blood phase. Due to the higher resolution of the PET images, it was possible to contour three-dimensional volumes of interest on both cortical and medullary segments and obtain time activity curves (**Figure 3**) 14. Compared to conventional scintigraphy, PET/CT information along with 3D data may allow for a more accurate analysis of renal function independent of background accumulation, renal malformations, and rotation. As alluded to earlier, ^18^F has a higher positron yield (^18^F, 96.86% vs ^68^Ga, 89.14%) and lower positron energy (^18^F, 633 keV vs ^68^Ga, 1,899 keV) which increases diagnostic yield relative to ^68^Ga 8. Although the first-in-human results are encouraging 14, the protocol needs to be refined and validated in a larger number of patients to confirm these preliminary findings.

#### ^68^Ga-PSMA-11

Recently, split renal function has been evaluated by a ^68^Ga-labeled prostate-specific membrane antigen (PSMA) PET imaging agent. When compared to ^99m^Tc-MAG3 in 97 prostate cancer patients, high correlation indices for assessing split renal function between both techniques could be achieved 40.

#### ^68^Ga-Ga-HBED-CC-DiAsp

^68^Ga-Ga-HBED-CC-DiAsp (Di-Aspartic acid derivative of N,N'-bis [2-hydroxy-5-(carboxyethyl)benzyl]-ethylenediamine-N,N'-diacetic acid) is a kit-based radiotracer, that potentially exhibits a higher stability when compared to 68Ga-EDTA, rendering it as a potential diagnostic agent to assess GFR *in-vivo*. In a rat study, biodistribution and dynamic PET/CT imaging studies in rats revealed a rapid clearance 41.

### ERPF agents

#### p-[^18^F]Fluorohippurate (^18^F-PFH)

^18^F-PFH reflects ERPF and may predict the longterm prognosis of the kidney as demonstrated in animal studies 42. The molecular structure of ^18^F-PFH is similar to that of *p*-aminohippurate, a gold standard for the measurement of ERPF. Awasthi et al. reported the synthesis and initial *in vivo* evaluation of ^18^F-PFH in a rat model. Dynamic PET revealed a rapid clearance of ^18^F-PFH through the renal-urinary pathway 43. In light of these encouraging findings, Pathuri et al. compared the renogram of ^18^F-PFH with ^125^I-OIH as a gold standard, and with a clinically used agent (^99m^Tc-MAG3), in healthy rats. The results showed no significant difference in time-to-peak, but in the visual analysis of renograms, ^18^F-PFH seemed to be closer to ^125^I-OIH compared to ^99m^Tc-MAG3. In addition, the quality of the renogram and the images obtained with the dynamic ^18^F-PFH PET study were remarkably better than those obtained with the ^99m^Tc-MAG3 dynamic planar imaging study 44. Additionally, the same research group evaluated the potential of early ^18^F-PFH PET renography to predict future progression of PKD in Han:SPRD rats with slowly progressive autosomal dominant PKD. They obtained T2 and T20 values, which represent the percentage of the injected dose of ^18^F-PFH in kidneys at 2 and 20 min after injection, from the imaging data and assessed T20/T2 ratio as a prognostic marker. T20/T2 values at 6 weeks correlated with serum creatinine, serum urea nitrogen, and kidney weight per body weight at 26 weeks. These results demonstrated ^18^F-PFH could be a surrogate marker, e.g. for progression of autosomal dominant polycycstic kidney disease 42.

#### Re(CO)3(^18^F-FEDA)

Re(CO)3(^18^F-FEDA) is an ERPF agent, and can be labeled with either ^99m^Tc or ^18^F, depending on the application. Lipowska et al. demonstrated that ^99m^Tc-Re(CO)3(FEDA) had comparable pharmacokinetics to ^131^I-OIH in rats 45 and its ^18^F-labeled counterpart exhibited a high specificity for the kidneys along with rapid renal excretion and high *in vitro* and *in vivo* stability comparable to those of its ^99m^Tc-labeled analogue 46.

#### ^11^C-PABA

**Figure 4:**
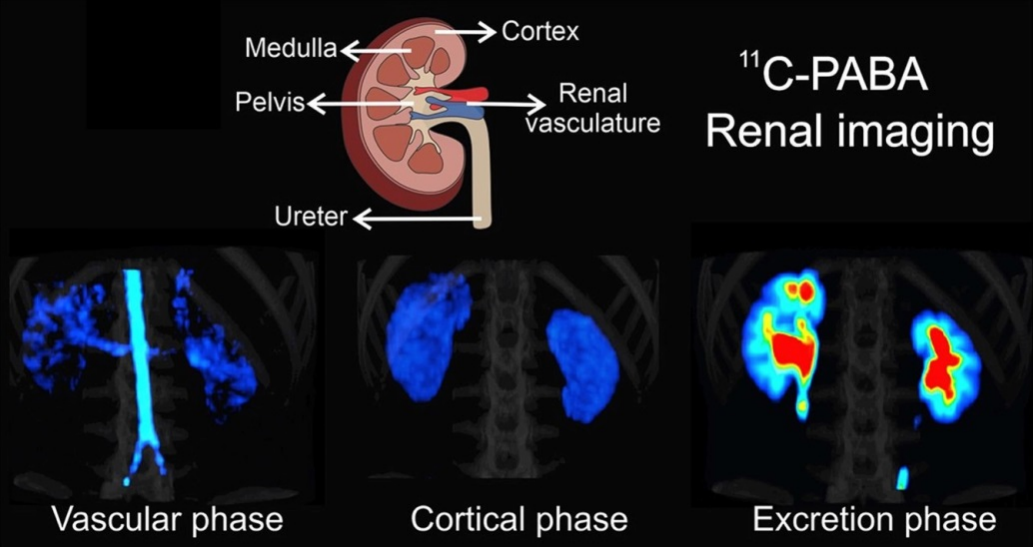
**^ 11^C-para-Aminobenzoic acid (^11^C-PABA) PET/CT renal imaging in a healthy subject.** After a vascular phase after 22 seconds after injecvtion, the cortical uptake was evident within 2.7 and 3.2 minutes, with maximal parenchymal uptake in the renal cortex. The excretory phase was seen after 9 minutes indicating the maximal uptake in the pelvicalyceal system. Modified from Ruiz-Bedoya et al 12, copyright 2020 by the Society of Nuclear Medicine and Molecular Imaging, Inc.

Most recently, Ruiz-Bedoya et al. investigated ^11^C-PABA as a new PET renal agent. PABA is a non-toxic B-complex vitamin with fast renal excretion, and has been previously used to assess 24-hour urine tests. One of its main metabolites, *para*-aminohippuric acid, has been defined as a gold standard to measure ERPF due to its high renal tubular secretion and thus, ^11^C-PABA may provide reliable information on ERPF. The authors confirmed the pharmacokinetics of ^11^C-PABA in rats and rabbits with dynamic PET, and performed first-in-human PET imaging studies in healthy volunteers. Dynamic PET demonstrated a rapid accumulation of ^11^C-PABA in the renal cortex, followed by rapid excretion through the pelvicalyceal system with low background signal from other organs in rats and rabbits. In humans, the cortex was delineated on PET, and the activity gradually shifted to the medulla and renal pelvis with high spatiotemporal resolution. In addition, the biodistribution of ^11^C-PABA and ^99m^Tc-MAG3 in postmortem tissues were directly compared by measuring organ-associated radioactivity 30 minutes after simultaneous injection of both radiotracers in identical rats. The results revealed a lower background activity in normal tissue for ^11^C-PABA compared to ^99m^Tc-MAG3. Moreover, relative to ^99m^Tc-MAG3, a human dose analysis based on mouse data for ^11^C-PABA showed that the latter radiotracer had a 1.6-fold lower dose. Although the short half-life requires an on-site cyclotron, which increases costs, the authors concluded that ^11^C-PABA could be used as a novel radiotracer for functional renal imaging, providing high-quality spatiotemporal images with low radiation exposure, rendering ^11^C-PABA as a suitable imaging agent for pediatric populations 12. **Figure 4** provides an overview of renal imaging using ^11^C-PABA.

### GFR and ERPF Assessments

#### ^18^F-FDG

Attempts to calculate renal function using ^18^F-FDG, which is most commonly used in daily clinical practice, have also been reported. Although it appears demanding to obtain information about renal function from a substance like ^18^F-FDG which is involved in various physiological processes, such an approach would be of great benefit, in particular as information on renal function could be obtained in addition to other clinical applications, e.g. tumor staging. In this regard, Geist et al. performed dynamic ^18^F-FDG-PET/MRI and Patlak analysis to determine GFR and ERPF in 24 healthy volunteers. Using blood-based creatinine clearance and the ^99m^Tc-MAG3 tubular extraction rate as a reference, total kidney GFR and ERPF as estimated by dynamic PET/MRI were highly correlated when compared to the reference standard (r=0.82-0.88) 47. Apart from ^18^F-FDG, ^11^C-acetate PET/CT may also provide human glomerular filtration rate based on compartmental modeling 48.

### Renal Blood Flow

**Figure 5:**
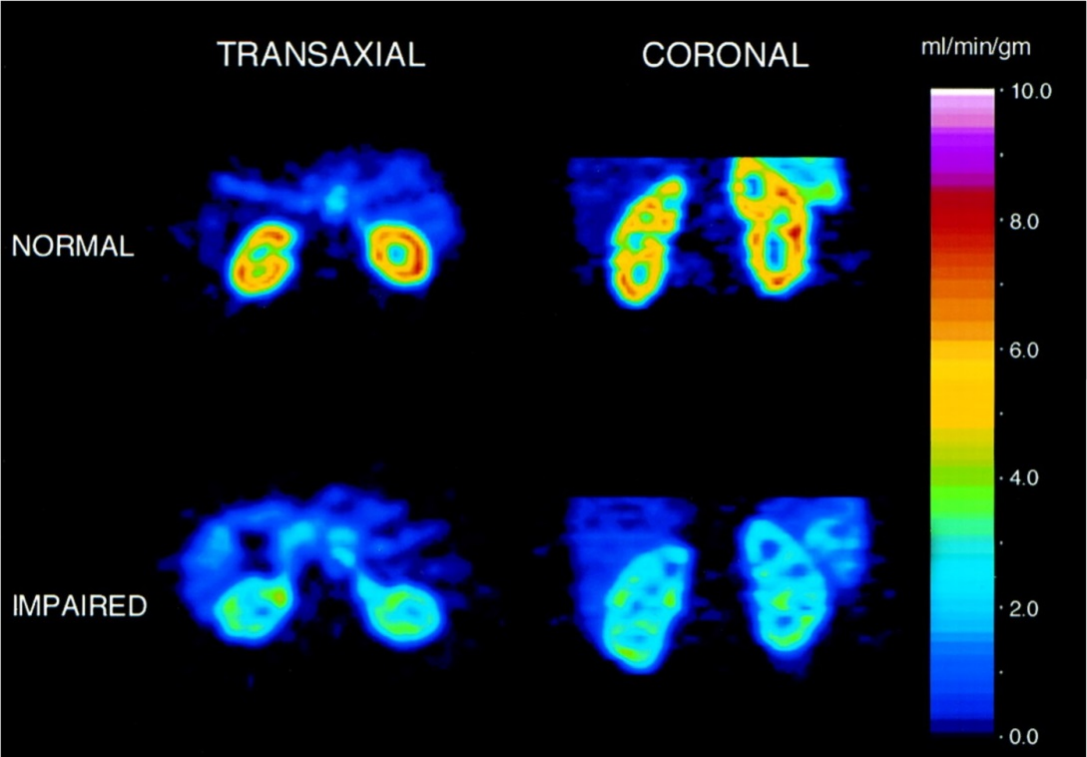
Renal blood flow assessed by H_2_^15^O PET of a healthy subject (*upper row*) vs. subject with moderate renal disease (*lower row*). Left: transverse view, right: coronal views. Modified from Alpert et *al* 49, copyright 2002 by the Society of Nuclear Medicine and Molecular Imaging, Inc.

Several radiotracers have been introduced for assessing renal blood flow (RBF). For instance, H_2_^15^O, which is freely diffusible between tissue and blood, has been investigated in patients with renal dysfunction demonstrating significantly lower flow values when normalized to 100 g of tissue measured with H_2_^15^O when compared to healthy individuals (**Figure 5**) 49. Owing to its short half-life of 2.1 min, more practical radiopharmaceuticals for reliably assessing RBF are intensively sought. In this regard, another class of RBF measuring agents has penetrated the clinical arenas, which are physiologically retained in renal tissue 50. Among others, ^82^Rubidium (^82^Rb) has a high first pass extraction and slow renal washout rendering it as an ideal imaging agent to measure RBF. Tamaki et al investigated ^82^Rb in dogs using a steady-state kinetic model with various degrees of kidney injury and concluded that ^82^Rb allows for serial quantitative assessment of RBF 51. This has been further evaluated for clinical use by investigating healthy volunteers showing that non-invasive quantitative imaging with ^82^Rb is feasible, allowing for excellent image quality, high resolution and contrast 52. In addition, this radiotracer is a potassium analogue and therefore, a recent report also demonstrated that renal potassium excretion can be visualized by ^82^Rb PET 53. Moreover, ^13^N-ammonia, which is widely utilized for myocardial perfusion imaging 54, has been also investigated to quantify RBF 55, which paves the way for assessing cardiorenal perfusion crosstalk. Of note, the renal extraction of this radiotracer is 100% and RBF obtained by ^13^N-ammonia demonstrated an excellent correlation when compared to the reference standard H_2_^15^O 56.

### Renal Inflammation Molecular Imaging

**Figure 6:**
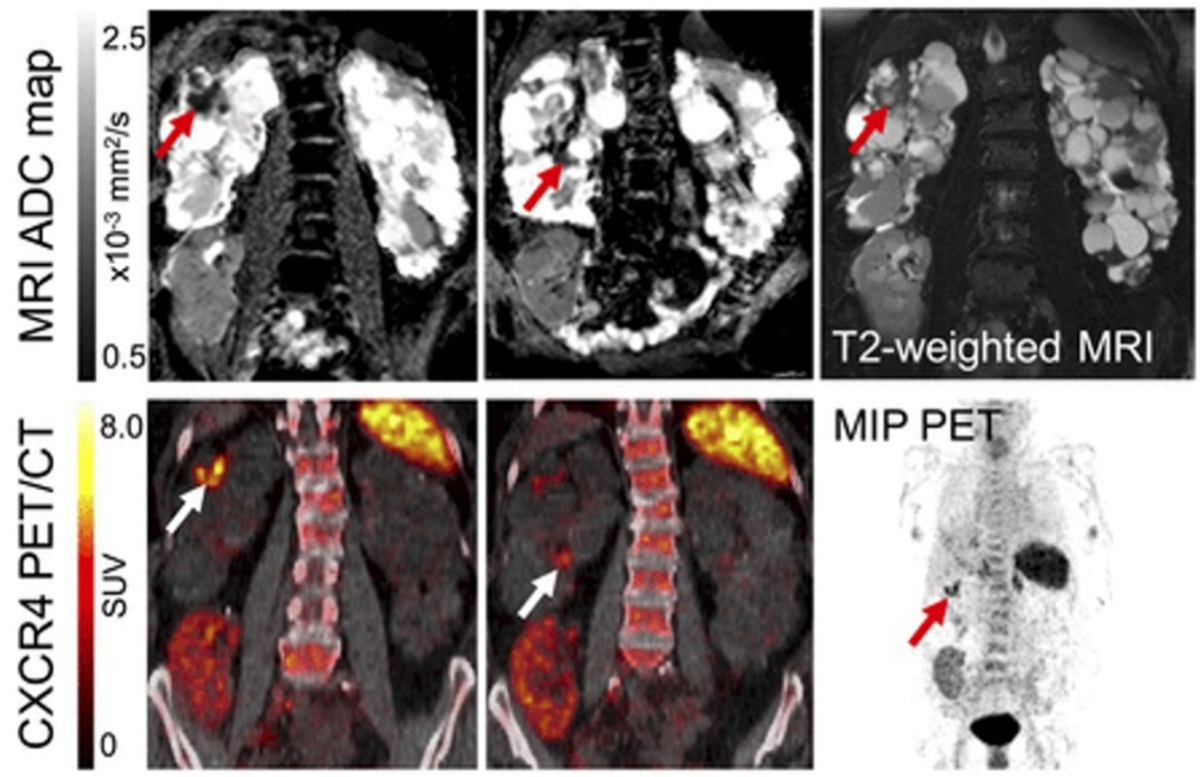
** PET and MR of acute renal cyst infection. MR (top) and PET/CT images (bottom) of patient with kidney transplantation due to autosomal-dominant polycystic kidney disease and suspected complicated urinay tract infection.** Imaging revealed upregulated CXCR4 expression in 2 cysts (white arrow) showing renal cyst infection. Corresponding MRI confirms a cyst with thick wall (T2-weighted MRI, red arrow) and apparent diffusion coefficient (ADC) reduction in both areas, consistent with PET findings. Allograft in right lower abdomen, however, shows no signs of infection with low homogeneous ^68^Ga-Pentixafor signal. MRI shows linear ADC reduction and volume loss, consistent with scarring at upper pole. MIP = maximum-intensity projection. Modified from Derlin et al 16, copyright 2017 by the Society of Nuclear Medicine and Molecular Imaging, Inc.

A recent study evaluated the value of ^18^F-FDG for autosomal dominant polycycstic kidney disease in 30 individuals and revealed a high diagnostic accuracy in detecting renal or hepatic cyst infections. Highlighting the potential of guiding therapy, baseline ^18^F-FDG PET/CT led to change in antiobiotics use in up to 63% of the patients 57. ^18^F-FDG, however, is rather nonspecific for inflammation imaging. ^68^Ga-Pentixafor targeting the C-X-C motif chemokine receptor 4 (overexpressed on leucocytes) has been investigated in patients with complicated urinary tract infections in combination with rMRI. Leukocyte infiltration was identified by areas of CXCR4 upregulation compared with unaffected parenchyma in PET, corresponding to areas with increased cell density in rMRI, which was further validated by *ex vivo* analysis. As such, combined PET/rMRI data revealed the potential of non-invasive detection of leukocytes in renal allografts 16 (**Figure 6**).

### Molecular Imaging of Renal Cell Carcinoma

Assessment of primary renal tumors is challenging, mainly due the high physiological uptake seen with imaging agents such as ^18^F-FDG or somatostatin receptor-targeting ^68^Ga-DOTA-D-Phe-Tyr3-octreotide (DOTATOC) or ^68^Ga-DOTA-D-Phe-Tyr3-octreotate (DOTATATE) 58. The United States Food and Drug Administration (FDA) approved radiotracer Anti-1-amino-3-[^18^F]-flurocyclobutane-1-carboxylic acid (anti-^18^F-FACBC) assessing the upregulation of increased amino acid metabolism, however, has almost no renal excretion. Spearheaded by Schuster et al, six patients afflicted with renal lesions were investigated and correlation with histology revealed that amino acid transport compared with renal cortex is elevated in renal papillary cell carcinoma but not in clear cell renal cell carcinoma (ccRCC) 59. Similar results have been gained from a rapid autopsy study investigating the second-generation prostate-specific membrane antigen (PSMA) agent ^18^F-DCFPyL demonstrating an accurate detection of sites of metastatic ccRCC 60. In addition, lymph node diseases along with photopenic defects in the kidneys have been recently reported in a biopsy-proven oncocytoma using ^18^F-DCFPyL 61. The ^68^Ga-labeled imaging agent PSMA-11 has received FDA approval just recently, which makes PSMA PET imaging more widely available for potential use in RCC. Nonetheless, such radiopharmaceuticals have been primarily investigated for assessing tumor burden in patients afflicted with RCC, whereas their use for renal functional imaging is rather limited 40.

### Clinical Applications

Although the use of PET can be limited by the installed base and high synthesis and maintenance costs, there are definite clinical applications of new renal PET radiotracers. For example, the low radiation exposures may lead to use in pediatrics, and the intrinsic advantages of PET may make these agents useful for those settings where an accurate renal functional assessment is required. Measurement of renal function in children can be difficult with noninvasive techniques because changes in body size and creatinine excretion can make creatinine clearance less reliable. In addition, children often have ureteral obstruction and anatomic abnormalities which may not be well evaluated by convetional scintigraphy 18. Although there are many hurdles to overcome, renal PET, with its anatomic information and high resolution, may address those issues.

In pediatric nuclear medicine, nuclear medicine specialists are facing the dilemma of reducing both radiation exposure and scan time. According to an IAEA study of 133 institutions from 62 countries on pediatric renal scintigraphy, most of the institutions would in fact administer a higher activity as recommended. The authors noted that the sites may have prioritized a shorter scan time (balanced by higher injected activities) rather than reducing radiation exposure 62. Given the higher count rates achieved with renal PET imaging relative to conventional scintigraphy, the administered activity could be significantly reduced for renal PET radiotracers while not sacrificing image quality or scan time. For example, Hofmann et al. typically administered 40 MBq of ^68^Ga-EDTA in adult patients, which results in approximately 1.6 mSv from the PET component and equates to approximately the exposure of 320 MBq of injected ^99m^Tc-DTPA 11. In addition, Ruiz-Bedoya et al. demonstrated that the effective dose of ^11^C-PABA was 1.6-fold lower than that of ^99m^Tc-MAG3 in a murine data-based human dosimetry analysis. The CT component of the PET/CT would add additional radiation exposure to the patient, but this can be as low as 0.25 mSv with an ultralow dose CT and the limited field-of-view required for renal imaging 10-12. As a result, renal PET minimizes radiation exposure, which is especially important for patients in need for an accurate assessment of renal function, e.g. while under chemotherapy or to monitor outcome after kidney transplantation 9, 15, 18. Renal PET could also be an alternative to CT-based urography to investigate urinary tract obstruction, especially for patients who are allergic to iodine contrast agents 11, 15.

### Future perspectives of PET in the field of nephro-urology

#### Identifying high-risk cancer patients for later nephrotoxicity under anti-tumor regimens

Recent years have also seen an expanded use of renal radionuclide SPECT imaging to identify patients which may have renal functional decline after multiple cycles of endoradiotherapies. There has been an expansion of the use of receptor radionuclide therapy (PRRT) in neuroendocrine tumors using somatostatin analogs labeled with beta-emitting radionuclides 63. However, PRRT causes an impairment of renal function even years after initiation of therapy and, therefore, pre-therapeutic and serial measurements of kidney function have been recommended 64. Of note, serum creatinine-based GFR estimates are insensitive to early toxicity and underestimate renal impairment in 12% of subjects after PRRT as compared to GFR measured by ^99m^Tc-DTPA 65-67. Although renal irradiation from PRRT is most likely to be caused by uptake of radiolabeled somatostatin analogues in the proximal tubules, ^99m^Tc-MAG3 reflecting tubular function could not identify high-risk patients with a late onset of renal failure 67. Furthermore alpha-emitters (such as ^223^Ra and ^225^At) are now being used clinically, and their higher mean energy deposition and shorter range in tissue relative to beta radiation may allow for increased therapeutic efficacy with minimal side effects in normal tissue 68. In this regard, Kratochwil et al. reported on two patients afflicted with metastatic prostate cancer, who received ^225^Ac-PSMA 69, while Makvandi et al. observed increased renal toxicity with^ 225^Ac and ^213^Bi in preclinical studies 70. Not surprisingly, renal toxicity with ^213^Bi-DOTATATE for treatment of neuroendocrine tumors has also been reported 71. In addition, a substantial portion of prostate cancer patients scheduled for radioligand therapy with ^177^Lu-prostate-specific membrane antigen (PSMA) also develop renal decline 72. Thus, given the increased use of endoradotherapies for various cancers, PET agents for renal function may be superior for kidney outcome prediction when compared to clinical biomarkers of renal decline or conventional SPECT imaging. Beyond such nuclear medicine therapies, kidneys are also prone to damage caused by iodine contrast agents and chemotherapy. Thus, PET agents such as Re(CO)3(^18^F-FEDA) and ^18^F-PFH, which reflect proximal tubular secretion, may also provide a surrogate marker of short- and long-term renal damage under chemotherapeutic regimens 9.

#### Theranostics and organ network analyses in nephrology and urology

**Figure 7:**
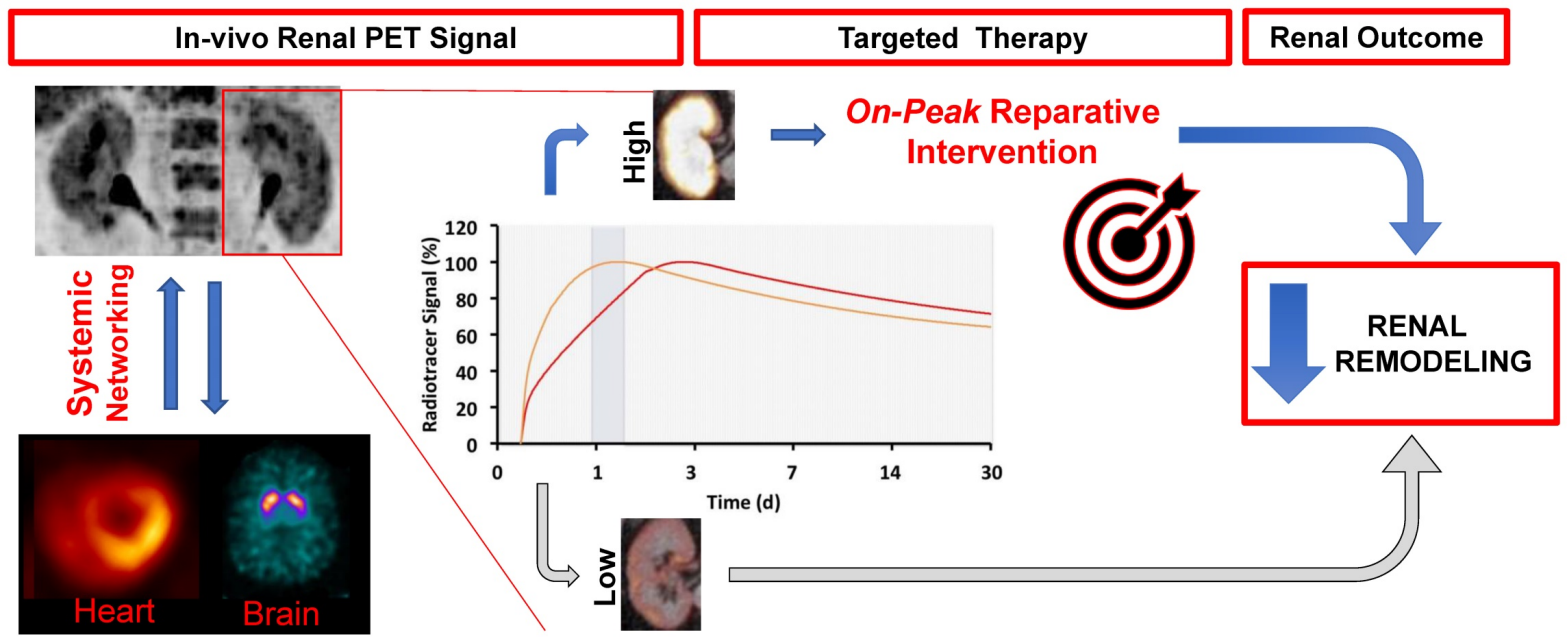
** Targeted and timed therapy for clinical applications in nephrology by using molecular imaging (theranostics).** By using dedicated radiotracers, e.g. to assess inflammation in the kidneys, a targeted therapy for an *on-peak* reperative intervention (anti-inflammatory therapy) could be initiated at the maximum of target expression. Both the diagnostic and matched therapeutic agent use the identical or at least comparable molecular pathways. Such a molecular imaging-guided treatment strategy for identifying the ideal time point for on-peak treatment may prevent adverse renal remodeling. In addition, a follow-up PET/CT to re-evaluate treatment efficacy can be considered. The derived imaging signal at baseline may be also a useful predictor for outcome, with defined endpoints such as major adverse renal events (MARE). Moreover, as PET allows for a whole-body non-invasive read-out, interactions of the kidneys with other organs, such as spleen, bone marrow or vessel walls could be conducted.

The theranostic concept in oncology is based on diagnostic molecular imaging, followed by an individually tailored treatment decision using the identical or similar therapeutic radiotracer 58. In nephro-urology, targeted and timed therapeutic concepts could also be envisaged for clinical applications for the kidneys. Such an image-guided renoprotective strategy would identify dysregulation of the target mechanism in the kidneys (e.g. upregulation of inflammatory-related mediators in urinary tract infections), which indicates risk for adverse outcome. This may trigger targeted therapy assessing the identical target on a subcellular level in order to achieve favourable renal outcome. For instance, receptor blockade of CXCR4 using AMD-3100 increased bladder capacity and hyperexcitability in bladder-inflammed Wistar rats relative to controls 73. As such, an inflammation-targeted read-out of the urinary tract system by using ^68^Ga-Pentixafor may identify the most suitable time-point for treatment initiation, which in turn may increase therapeutic efficacy (**Figure 7**). In addition, the baseline-derived radiotracer signal may be used as a predictor of outcome with defined endpoints such as major adverse renal events (MARE).

In uro-oncology, PSMA-PET/CT assists in identifying prostate cancer patients which are suitable for PSMA-based radioligand therapy (RLT). This theranostic concept is currently penetrating the clinical arena, mainly due favorable results of recent major prospective trials. First, the phase 2 LuPSMA trial reported on progressive metastatic castration-resistant prostate cancer patients that have been previously treated with antiandrogens and taxane-based chemotherapy. RLT demonstrated high response rates and low toxic effects with grade 3 or 4 thrombocytopenia in 13% of the patients 74. These favorable results have been further confirmed in the TheraP trial reporting on higher PSA response and fewer grade 3 or 4 adverse events of RLT when compared with cabazitaxel 75. The VISION trial (NCT03511664) will assess the outcome of patients treated with RLT in addition to best supportive/best standard of care versus patients treated with best supportive/best standard of care alone 76. The LuPARP trial will investigate RLT in combination with the poly(ADP-ribose) polymerase (PARP) inhibitor Olaparib (NCT03874884) 77, whereas the PRINCE trial will investigate favorable effects of PSMA-targeted therapy with the PD1-checkpoint inhibitor pembrolizumab (NCT03658447) 78. Results of these trials will further refine the role of theranostics in men afflicted with prostate carcinoma in various clinical scenarios.

As alluded to earlier, PSMA-targeting imaging agents have also been investigated for potential use in RCC. In this regard, 16 patients with suspicion for ccRCC have been scanned with ^68^Ga-labeled PSMA PET/CT and 75% of the patients demonstrated avid primary lesions, with the majority of ccRCC subtype. Of note, in 42% of the cases, ^68^Ga-PSMA PET/CT led to change in management, due to the identification of new sites of suspected metastases and synchronous primaries 79. Beyond its potential role in guiding clinical management, PSMA may also represent a promising therapeutic target in a theranostic setting. First, given the increased intrarenal tumor neovasculature which is tightly linked to increased PSMA expression 80, PSMA imaging may allow for patient selection for antiangiogenic therapies. These considerations are further fueled by recent reports of a prospective trial investigating the beneficial effects of a combination of an immune checkpoint inhibitor with a VEGF-targeted antiangiogenic drug. The anti-programmed death ligand 1 (PD-L1) antibody avelumab was combined with the highly selective vascular endothelial growth factor (VEGFR) inhibitor axitinib in patients with previously untreated advanced RCC. When compared with sunitinib, progression-free survival was significantly prolonged when used in first-line treatment, but grade 3 adverse events still occurred in 71% receiving such a combination treatment 81. Multiple trials of antiangiogenic drugs targeting the VEGFR pathway are currently ongoing and resistance to antiangiogenic therapy in clinical practice seems to be a critical issue for future developments in the field. Therefore, PSMA-directed imaging could not only help to increase therapeutic efficacy by selecting individuals with increased intrarenal tumor-driven angiogenesis but may also avoid unnecessary treatments or led to early termination of VEGFR-interacting drugs, e.g. once the signal has been dissipated on restaging under antiangiogenic regimens 82. Second, aggressive RCC subtypes with increased PSMA expression on the tumor cell surface may also allow for a theranostic approach by identifying potential therapy candidates by ^68^Ga/^18^F-PSMA imaging followed by ^177^Lu-PSMA treatment 83. For instance, Spatz et al evaluated the relationship between PSMA expression and clinicopathological features in 257 patients with RCC, ccRCC, papillary RCC and chromophobe RCC. Of note, 82.5% of ccRCC and 71.4% of chromophobe RCC specimens demonstrated increased PSMA expression, whereas only 13.6% of papillary RCC expressed PSMA. Most importantly, the association of PSMA expression with overall survival was superior when compared to established clinical parameters, including tumor grade, primary and metastasis stage 84. In addition, RCC still has a poor prognosis, despite recent efforts of applying novel immune checkpoint inhibitors 85. If PSMA expression, however, increases with more aggressive disease, one may speculate that the signal derived by PSMA-targeting PET will not be diminished, even in highly advanced disease 84. Moreover, in chromophobe RCC, where therapeutic options are still rather limited, PSMA was also upregulated in the majority of the cases, supporting the notion of PSMA-based theranostics in this rare phenotype 83. Last, efficacy of immunotherapy could be further enhanced by combination treatments with PSMA-targeting RLT in PSMA-positive RCC patients 51.

Apart from applying theranostics in nephrology and urology, interactions with remote organs, such as the vessels or the spleen, could be also pursued by whole-body molecular imaging, potentially allowing for non-invasive analyses of kidney-organ networks 86. Long-bore cameras with lager field-of-views and recent installations of whole-body PET scanners will further enable for such crosstalk investigations along various kidney-organ axes (**Figure 7**) 87. Such investigations may be of interest in the field of inflammatory renal molecular imaging, e.g. to assess the signal in organs serving as reservoirs of inflammatory cells, such as the bone marrow or kidneys. Other aspects may also include kidney interactions with the molecular brain signal, potentially allowing for a simultaneous read-out of renal and brain function. Further improving the PET-based read-out of the kidneys, dedicated protocols could be applied, e.g. by furosemide injection enabling for sufficient excretion of the radiotracer prior to the scan 16.

## Conclusions

Despite the development of CT, MRI, and ultrasound techniques for evaluating the kidney, renal scintigraphy is still frequently requested in clinical practice. In a manner similar to brain, cardiac, and tumor molecular imaging, renal radionuclide imaging is beginning a shift from SPECT to PET. The GFR-reflecting radiotracer ^18^F-FDS can be synthesized from ^18^F-FDG in a one-step synthesis and thus, ^18^F-FDS would be available at virtually every PET practice with access to a cyclotron. In addition, the relatively long half-life of ^18^F renders it as a potential agent for clinical trials, in particular in pediatric populations as it has significantly less radiation exposure compared to SPECT equivalents 9, 13, 14. Moreover, inflammatory-targeting radiotracers have been investigated for renal applications, e.g. in patients with urinary tract infections. This may open avenues for timed and targeted therapies at the maximum of the PET-derived target expression, potentially transferring the concept of theranostics from oncology to nephron-urology. Moreover, given the capability of PET for a whole-body systemic read-out, such a molecular image-guided strategy could be used for a wholistic benefit not only maximizing outcome in the target organ (kidneys) but also in remote organs. Nonetheless, whole-body PET scanners are currently sparsely available. Commonly used hybrid devices with 16 cm field of view may still allow for assessing split renal function and clearance measurements. In addition, a more widespread adoption of PET radiotracers for renal applications is needed, preferably in prospective clinical trials. Favorable results relative to conventional SPECT would also pave the way for FDA approval of renal functional PET radiotracers, which would then lead to an increased clinical use of PET for this purpose.

## Figures and Tables

**Table 1:** Characteristics of renal scintigraphy along with clinical indications.

Categories	Filtered by the glomerulus	Secreted by the tubules	Tubular fixation
**Single photon radiotracers**	^99m^Tc-DTPA	^99m^Tc-MAG3	^99m^Tc-DMSA
**Main indication**	Measurement of GFR	Measurement of ERPF	Morphological assessment
**Alternate in clinical practice**	Serum creatinine, Creatinine clearance, Inulin clearance	PAH Clearance	Ultrasound, etc.
**Other clinical indication**	Obstructive uropathy, Renal transplant rejection, Renovascular hypertension		Pyelonephritis and parenchymal scarring

DTPA: diethylenetriaminepentaacetic acid, MAG3: mercaptoacetyltriglycine, DMSA: dimercaptosuccinic acid, GFR: glomerular filtration rate, ERPF: effective renal plasma flow, PAH: p-aminohippurate.

**Table 2:** Head-to-head comparison of different image modalities for assessing renal function.

Imaging Modality	MRI	CT	Ultrasound	PET
**Advantages**	Allows for precise tissue differentiation 88 Can examine various physiologic aspects of renal function (perfusion, glomerular filtration, interstitial diffusion, tissue oxygenation, tubular transit) 88 MR renography can reliably diagnose renovascular hypertension, urinary obstruction or renal transplant complications 88 Assessment of split-renal function 89 Better arterial input functions and separation in duplicate kidneys relative to PET 89 Can compensate motion (kidneys are prone to motion artefacts given their anatomical localization under the diaphragm) 89	Widely available Various clinical applications, including staging/restaging, obstruction (kidney stones, polycystic kidney disease) Localizing of abscesses Preoperative planning prior to partial nephrectomy 89	Virtually available at every hospital 90 Low costs 90 No need for intravenous iodine contrast adminis-tration 90 Fast read-out of kidneys and adjacent organs, e.g. for renal cyst evaluation	Large variety of renal function can be assessed, e.g. GFR, ERPF, inflammation, RBF or renal PSMA expression 3-dimensional read-out capabilities Assessment of split-renal function 1mSv radiation exposure 89 No pharmalogical effects 89 Can be used in a theranostic setting, e.g, for RCC or to assess organ-organ interactions
**Disadvantages**	Confined space and loud noises leading to patient discomfort Certain devices, such as pacemakers, are harmful Risk of nephrogenic systemic fibrosis with MRI contrast agents 91 Spatial resolution is limited as only multiple 2D slices are provided 89	Life-threatening contrast-induced nephropathy with CT contrast agents 91 Anaphylactoid reactions CT contrast media may lead to drug-drug interactions, e.g. increasing the retention of metformin leading to lactic acidosis 91 Radiation exposure (up to 8 mSv) 89	Spatial resolution is limited with single 2D slices 89 Does not provide detailed functional information	PET radionuclides emit all the same energy photons at 511keV and therefore, it is not feasible to use multiple PET tracers at the same time 89 No larger clinical trials to date No FDA approval to date for PET radiotracers evaluating renal function

MRI: magnetic resonance imaging, CT: computed tomography, PET: positron emission tomography, GFR: glomerular filtration rate, ERPF: ERPF: effective renal plasma flow, PSMA: prostate-specific membrane antigen, FDA: Food and Drug Administration.

**Table 3:** Characteristics of positron emitters.

	^11^C	^18^F	^68^Ga
**Half-life (min)**	20	110	68
**Production source**	on site cyclotron	on site cyclotron or delivery	^68^Ge/^68^GaGenerator
**Positron energy (MeV)**	0.96	0.64	1.90
**Positron range (mm)**	1.1	1.0	2.9
**Renal PET tracer**	^11^C-PABA	^18^F-FDS ^18^F-PFH Re(CO)_3_^18^F-FEDA	^68^Ga-EDTA ^68^Ga-NOTA ^68^Ga-RDye800-tilmanocept
**Advantage**	Less exposure to radiation	Lower cost Delivery available Flexibility due to longer half-life Physical properties suitable for imaging	Can be operated without a cyclotron

PABA: para-aminobenzoic acid, FDS: 2-deoxy-2-fluoro-D-glucose, PFH: p-fluorohippurate Re(CO)3FEDA: Re(CO)3-N-(fluoroethyl)iminodiacetic acid, EDTA: ethylenediaminetetraacetic acid, NOTA: 1,4,7-Triazacyclononane-1,4,7-triacetic acid.
